# Mr. Peck, in Reply to Dr. Squire

**Published:** 1800-06

**Authors:** Thomas Peck

**Affiliations:** Higham Ferrars


### *To the Editors of the Medical and Phyſical Journal.*

GENTLEMEN,

THE very pointed manner in which Dr. Squire has animadverted on my opinion of "the expediency of an early delivery of the placenta," *naturally* engages my attention; which is the more excited from a ſuppoſition that he has conſiderably *perverted* my deſign. If, therefore, *as addenda*, you will give place, place, in your next, to the following remarks, you will particularly oblige,

GENTLEMEN,

Your's, refpectfully,

THOMAS PECK.

*Higham Ferrars,*

*May* 6, 1800.

---

THE firft idea which Dr. Squire attacks, is that of the propriety of an "immediate removal of the placenta." Now, it is no difficult tafk to *wrest* an opinion: For inftance, detach a fentence, and Scripture at once appears *falfe.* "There is no God." But, who has faid it?—I have urged, that the placenta cannot be too fpeedily removed after the expulfion of the child. Dr. Squire has, very uncharitably, omitted the connection; "I do not mean to urge the *propriety* of a *forcible* extraction of the placenta the very moment the funis is divided, but to pay *direct attention* to the efforts of Nature; and if *fuch* efforts are not fufficient to expel it in ten or fifteen minutes, to extract it;"—and I would, here, beg to be underftood, "not to pull with a force which may endanger its feparation."

Dr. Squire then fays, "To wait no longer than ten or fifteen minutes for the efforts of Nature, is *a pofition which cannot be too ftrongly reprobated, unlefs flooding, or other untoward accident, fhould require the affiftance of art.*" In Mr. Davies's cafe, however, the *former* took place; and this led me to fuppofe, that an immediate extraction of the placenta was requifite, as a means of preventing an hæmorrhage, which is *always* to be dreaded, and is not *unfrequently* fatal. Would Dr. S. have remained an idle fpectator in a cafe of hæmorrhage, *clearly* arifing from adhering placenta? Would he have remained inactive in a cafe of *confessedly* "*alarming*" *flooding*, when proper "manual operation" might have prevented its continuance? Certainly, he would not.—Dr. S. appears to deduce a felf-pleafing inference from the fortunate termination of Mr. Davies's cafe, by faying, "We may indulge hope in the moft *defperate* fituation, *this* inftance affording a proof of the ftrength and refources of the human conftitution." But, does *this* cafe *fufficiently* warrant a fimilar conduct in all fuch inftances? Mr. Pott, in his Remarks on Amputation, Vol. III. p. 362, has a paffage which may tend to illuftrate my idea: "When a judicious man fays that a limb *ought* to be removed, it is not to be fuppofed that he means to fay, that it is abfolutely impoffible, at all events, that fuch limb can be faved; nor, that fuch patient muft infallibly die, if the operation be not performed; no, he only means, *that from repeated* experience of himfelf, himſelf, and others, in all times, it has been found that the circumſtances above-mentioned put the patient's life much more to hazard in an attempt to ſave the limb, than the operation does in removing it; and, *therefore*, that humanity, as well as judgment, determine for the latter." As it reſpects the delivery of the placenta, I mean to ſay, in *caſes of urgency*, I would prefer the *extraction* of it to the waiting for the efforts of Nature.—Dr. Squire has thought fit to quote very reſpectable authorities in ſupport of his reaſonings: Firſt, Dr. Smellie: "If there is no danger from a flooding, the woman may be allowed to reſt a little, in order to recover from the fatigue ſhe has undergone, &c." A practice allowed to be conſiſtent; a practice I *have* plainly acceded to, by ſaying I would wait "ten or fifteen minutes;" and I would not *even confine* myſelf to that period, except in caſe of hæmorrhage, or other untoward circumſtance: I mean to ſay, that, *generally* ſpeaking, *in that time* the uterus will contract *ſufficiently* to throw down the placenta; but *even then*, how often do we find it diſpoſed to remain in the vagina, unleſs it be removed per artem. The next extract is from Dr. Hunter's Lectures, "Whether the placenta comes in a few minutes, or an hour, uſe little or *no force*, &c." I *perfectly agree* with *this* practice *when I preſs* the *impropriety* of a "*forcible*" extraction of it:—nor have I *ever* found a *neceſſity* for any *exertion*, when the placenta has *not* been retained longer than ten or fifteen minutes; but I have *frequently* been called to caſes where the child has been expelled one, two, or more hours, wherein great difficulty has actually occurred.—Dr. Harvey is then quoted: He ſays, "by pulling down the burden by the navel-ſtring, *if a portion* is *ſtrongly* adherent to the uterus, we may by *this* force invert the uterus." But who, in ten or fifteen minutes after the birth of the child, would *forcibly* pull down by the funis? If I underſtand Dr. Harvey, in this place, he intends by it, that he would rather bring away the ſubſtance of the placenta by a graſp, than *(in caſes of retenſion)* depend on the firmneſs of the funis.—Dr. Squire, fourthly, has recourſe to Dr. Denman, who (after ſpeaking on the propriety of leaving the placenta to the action of the uterus) ſays, "We are at liberty to act when Nature is not ſufficient, or when *dangerous* circumſtances demand our aſſiſtance." Is not hæmorrhage a dangerous circumſtance? Dr. Denman goes on, "The *mere debility* of the patient is therefore *often* a reaſon why we ought to wait, without making any attempts to haſten the ſeparation or extraction of the placenta; as an immediate ſeparation, natural or artificial, would be an addition to the danger which ſhe was before in." I preſume it is unqueſtionably queſtionably intended, in *this paragraph*, where the *debility* ariſes from *fatigue* in labour, and not from profuſe hæmorrhage. We *next* have an extract from Mr. White: "Certain pain and danger *muſt* attend the operation; and, in *almoſt every caſe*, the odds are great but it is totally unneceſſary, &c." But Mr. White does *not* ſay it is inexpedient in *every caſe*. He knows inſtances where patients have materially ſuffered through the long detenſion of the placenta. Hamilton's Outlines are then ranſacked, and we find that "the introduction of *the hand* is *ſeldom* neceſſary, and never ſhould be had recourſe to except in the moſt urgent caſes." This meets my moſt cordial approbation. Dr. Oſborne ſays, "The natural expulſion of the placenta is both eaſier and ſafer than the artificial extraction, however ſkilfully performed." I know *no* practitioner who will dare to deny ſo plain a fact. Drs. Clarke and Bland are, laſtly, brought to prove, what every practitioner muſt know, that "the placenta ſhould *not* be delivered in an *haſty* manner; and that it very rarely is detained." Perhaps, the motto "*Naturâ monſtrante viam*," is as deeply impreſſed in my breaſt as in that of Dr. Squire; and, I aſſure him, it would be my *laſt* intention to counteract her benevolent dictates; and *however erroneous my* opinions in *practice* may appear to Dr. Squire, ſo *long* as they are not in *my* own view *irrational or dangerous*, ſo long ſhall I heartily adopt them: and I am extremely happy in noticing Dr. Scott's ideas as conſonant with *my own*, as well as the *experience* of many veterans in midwifery. Having, I hope, ſufficiently explained myſelf, and ſatisfactorily proved it is not my aim to miſlead, I leave the ſubject, *at preſent*, earneſtly wiſhing the conduct of Dr. Squire, towards parties entirely unknown to him, may, in future, be diveſted of aſperity.

